# The Role of Perineuronal Nets in Physiology and Disease: Insights from Recent Studies

**DOI:** 10.3390/cells14050321

**Published:** 2025-02-20

**Authors:** Sophia Auer, Martin Schicht, Lucas Hoffmann, Silvia Budday, Renato Frischknecht, Ingmar Blümcke, Friedrich Paulsen

**Affiliations:** 1 Institute of Functional and Clinical Anatomy, Friedrich-Alexander-Universität Erlangen-Nürnberg, 91054 Erlangen, Germany; martin.schicht@fau.de; 2 Department of Neuropathology, Universitätsklinikum Erlangen, Friedrich-Alexander-Universität Erlangen-Nürnberg, Partner of the European Reference Network (ERN) EpiCARE, 91054 Erlangen, Germany; lucas.hoffmann@uk-erlangen.de (L.H.); ingmar.bluemcke@uk-erlangen.de (I.B.); 3 Institute of Continuum Mechanics and Biomechanics, Friedrich-Alexander-Universität Erlangen-Nürnberg, 91058 Erlangen, Germany; silvia.budday@fau.de; 4 Department of Biology, Animal Physiology, Friedrich-Alexander-Universität Erlangen-Nürnberg, 91058 Erlangen, Germany; renato.frischknecht@fau.de

**Keywords:** extracellular matrix, perineuronal nets, schizophrenia, Alzheimer’s disease, epilepsy

## Abstract

Perineuronal nets (PNNs) are specialized extracellular matrix structures that predominantly surround inhibitory neurons in the central nervous system (CNS). They have been identified as crucial regulators of synaptic plasticity and neuronal excitability. This literature review aims to summarize the current state of knowledge about PNNs, their molecular composition and structure, as well as their functional roles and involvement in neurological diseases. Furthermore, future directions in PNN research are proposed, and the therapeutic potential of targeting PNNs to develop novel treatment options for various neurological disorders is explored. This review emphasizes the importance of PNNs in CNS physiology and pathology and underscores the need for further research in this area.

## 1. Introduction

In the central nervous system (CNS), neural cells are embedded within the extracellular space (ECS), a fluid-filled environment that contains the extracellular matrix (ECM), which serves as a structural scaffold for brain tissue [1]. During mammalian brain development, the ECS occupies approximately 40% of the total brain volume, decreasing to about 20% in adulthood [2,3]. The ECM is integral to the structural and functional integrity of the brain. Beside the “loose” matrix formed by the ECM, specialized forms of the ECM can be found, such as the so-called perineuronal net (PNN). Perineuronal nets (PNNs) are extracellular matrix structures that densely surround certain neurons in the central nervous system [4], e.g., fast-spiking inhibitory parvalbumin-expressing (PV+) GABAergic interneurons [5]. It has been shown that PNNs play a crucial role in regulating synaptic plasticity and neuronal excitability [6,7,8,9,10]. Their composition, including chondroitin sulfate proteoglycans (CSPGs), hyaluronan (HA), and linking proteins, contributes to the stabilization of synaptic contacts and protection of neurons from oxidative stress [11,12]. Furthermore, studies have suggested that alterations in PNNs may be involved in neurological disorders, such as epilepsy, Alzheimer’s disease, and schizophrenia [13,14,15,16]. Understanding the role of PNNs in the human brain is therefore essential for gaining insights into the pathophysiology of these conditions and potentially identifying novel therapeutic targets.

## 2. Distribution of PNNs in the Mammalian CNS

PNNs have mainly been observed in regions associated with sensory and cognitive function, such as the cerebral neocortex, the hippocampus, or the amygdala. However, PNNs have also been identified in the spinal cord [17]. Their distribution patterns vary within different mammalian species and brain regions, although most studies were conducted in rodents. The distribution pattern of PNNs and their colocalization with parvalbumin was systematically described for the adult mouse brain, showing an enrichment in layer IV and upper layer V of primary sensory cortex areas. In contrast, they were confined to layers II and III of the medial entorhinal cortex [18,19]. In the hippocampus, medial entorhinal cortex, and the visual cortex of rat and mouse brains, PNNs mainly surround fast-spiking PV+ inhibitory neurons. Beside inhibitory enwrapped neurons, PNNs were shown to be present around excitatory pyramidal cells in the hippocampal cornu ammonis 2 (CA2) region of mice [18,20]. Yamada and Jinno demonstrated the molecular heterogeneity of aggrecan-based PNNs in the mouse hippocampus, showing that several subclasses of PV+ GABAergic neurons are enwrapped in the CA1 and CA3 regions [21]. In the human brain, PNNs accumulate in layers III and V of the motor and somatosensory cortex, where they covered both non-pyramidal and pyramidal neurons [22]. In the human visual cortex, PNNs are also present in a subpopulation of PV+ interneurons, primarily in layers IV and VI of Brodmann area 17 (BA17) [23]. In the human hippocampus, PNNs were visible in all anatomical subfields (dentate gyrus, CA-subfields, and subiculum). However, the density varies within the anatomical regions and layers of the subregions [24]. The heterogeneity of PNNs across different species, brain regions, and neuronal populations adds complexity to the understanding and study of PNNs.

## 3. Molecular Structure of PNNs

**Figure 1:**
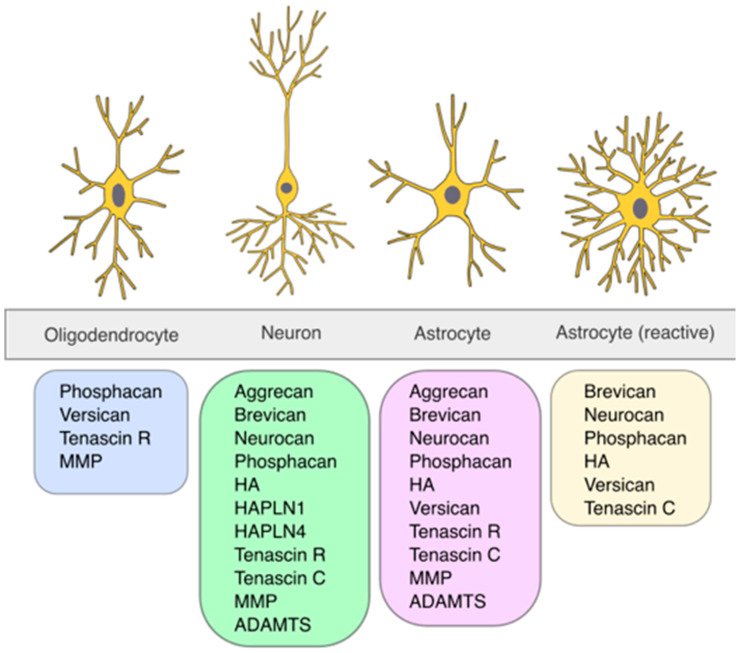
Cell-type-specific production of perineuronal net components. The figure illustrates the sources of various PNN components: oligodendrocytes [42,43,44,45], neurons [27,42,43,44,45,46,47,48,49,50], astrocytes [27,42,43,44,45,46,47,48,49,50], and reactive astrocytes [44,45,46,47,51]. Each cell type is represented with its characteristic morphology, accompanied by a list of the corresponding PNN components produced. Summarized in [39,52,53]. (Illustration by Jörg Pekarsky, Institute of Functional and Clinical Anatomy, FAU Erlangen-Nürnberg).

**Figure 2:**
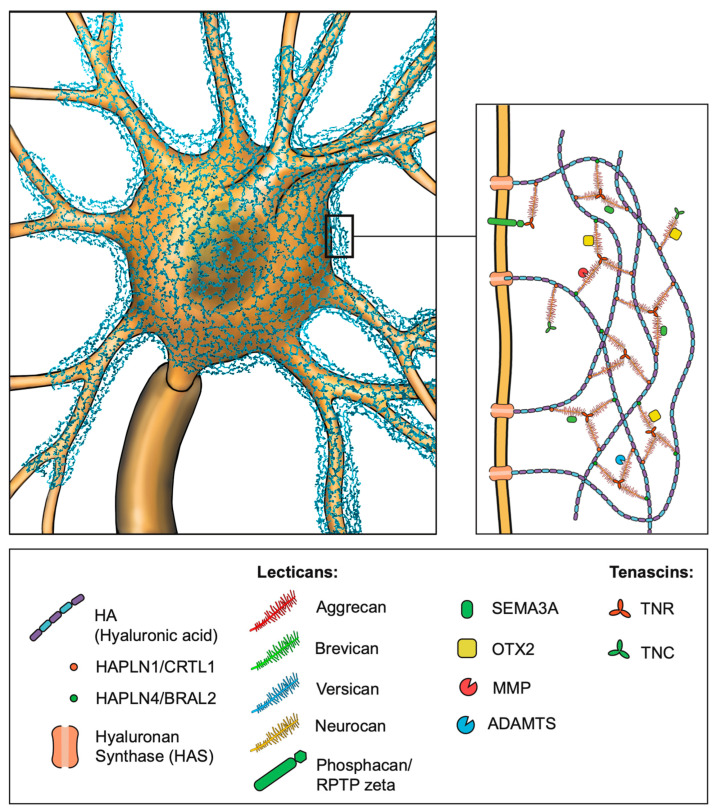
Schematic representation of the structure and composition of perineuronal nets (PNNs). The backbone of perineuronal nets is formed by hyaluronic acid (HA), which facilitates the binding of chondroitin sulfate proteoglycan (CSPG) lecticans via the link proteins HAPLN1 (CRTL1) and HAPLN4 (BRAL2). HA is produced by hyaluronan synthases (HASs) embedded in the neuronal membrane. The CSPG triads are further stabilized by tenascin-R (TNR), whereas tenascin-C (TNC) interacts with individual CSPGs. In addition to the HA attachment, CSPGs are linked to the cell membrane via interaction with the phosphacan/RPTPzeta receptor. The physiological remodeling of PNNs is mediated by two families of proteolytic enzymes: matrix metalloproteinases (MMPs) and a disintegrin and metalloproteinase with thrombospondin motifs (ADAMTSs). Additional molecules, such as OTX2 and SEMA3A, play a role in PNN maturation by binding to the glycosaminoglycan (GAG) side chains of CSPGs. Abbreviations: CSPG = chondroitin sulfate proteoglycans; HA = hyaluronic acid; TNR = tenascin-R; HAPLN = hyaluronan and proteoglycan link protein; ADAMTS = a disintegrin and metalloproteinase with thrombospondin motifs; OTX2 = orthodenticle homeobox 2; SEMA3A = semaphorin 3A; GAG = glycosaminoglycan; MMP = matrix metalloproteinase. (Illustration by Jörg Pekarsky, Institute of Functional and Clinical Anatomy, FAU Erlangen-Nürnberg).

PNNs were initially described in 1893 by Camillo Golgi [25]. However, their significance began to rise much later in the late 19th century as the techniques for studying PNNs became more advanced (for a detailed description of the discovery of PNNs, refer to [25]). PNNs are specialized forms of the ECM surrounding the soma, the axonal initial segment, and proximal dendrites of neurons. The major components of PNNs are hyaluronic acid (HA), chondroitin sulfate proteoglycans (CSGPs), tenascins, in particular, tenascin-R (TNR), and link proteins (CRTL1/HAPLN1 and BRAL2/HAPLN4), which are produced by various neuronal cell types (Figure 1). The hyaluronic backbone of the PNN provides structural stability and anchors the PNN to the cellular surface by interaction with the receptor CD44, expressed on both neurons and astrocytes [26]. HA is produced by hyaluronan synthases (HAS1–3) located at the membrane of the enveloped neuron [27]. Compared to the “loose” ECM, the PNNs display a denser structure with greater amounts of CSPG lecticans. These lecticans are produced by the surrounded neurons, but additionally by astrocytes or oligodendrocytes, and represent the organizers of the brain extracellular matrix with domains on the N- and C-terminals attached to the core protein [28]. The amino terminal regions facilitate binding to HA, while the carboxy terminal regions establish a connection with TNR. In addition to the globular domains, a varying amount of chondroitin sulfate glycosaminoglycan (GAG) is covalently bound to the core protein. Repeated chains of glucuronic acid (GlcA) and N-acetyl-galactosamine (GalNAc) form the structural basis of the GAGs. The number of GAG chains, the sulfation pattern of GlcA and GalNAc, and the core protein structure led to the identification of four CSPG lecticans: aggrecan, versican, brevican, and neurocan [29,30,31,32,33]. An additional CSPG is named phosphacan, a splice variant of the gene *PTPRZ1* encoding the receptor protein tyrosine phosphatase-zeta (RPTPzeta) [34]. Recently, it was shown that phosphacan may connect with TNR and function as an anchor for the PNN to the cellular surface, in addition to HA attachment [35]. The connection between CSPGs and HA is maintained by the link proteins cartilage link protein-/hyaluronan and proteoglycan link protein 1 (CRTL1/HAPLN1) and brain link protein-2/hyaluronan and proteoglycan link protein 4 (BRAL2/HAPLN4) [36,37]. The CSPGs are connected to each other in triads through TNR, which in turn contributes to the structural integrity of the PNNs [38,39]. Lastly, semaphorin3A (SEMA3A) and orthodenticle homeobox2 (OTX2) are molecules binding to GAG side chains and thereby controlling plasticity [40,41] (Figure 2).

## 4. Development and Plasticity

During brain development, the formation of PNNs occurs in a highly regulated manner, coinciding with critical periods of synaptic refinement and maturation that are unique to each brain region [54,55,56]. It has been shown that some of the main components of the PNNs are already expressed in the embryonic nervous system. However, their assembly into PNNs occurs later in development when the expression of PNN components is upregulated [57,58]. In the human developing brain, PNNs were shown to first be present in the second month of life, varying across different brain regions [57]. Their maturation is a gradual process, with them reaching complete maturation by the age of eight years [57]. In coincidence with this, parvalbumin (PV) was shown to be present at birth, but reaches its peak levels around the age of 2 years [57,59]. In addition, neuronal activity also has an impact on the PNNs’ formation. This was demonstrated by the fact that rearing rats without visual stimuli in the dark from birth prevented the formation of PNNs. As a result, reintroducing the animals into a normal light/dark environment could restore PNNs [60,61]. The maturation of the PNNs also depends on the expression of the two molecules SEMA3A and OTX2. Both play a crucial role in initiating and closing the critical period of plasticity in the developing brain [41,62,63,64]. The formation of PNNs is a dynamic process, and physiological remodeling persists even after the PNNs are fully matured. During formation and remodeling, the enzymes MMP (matrix metalloproteinase) and ADAMTS (matrix metalloproteinase and a disintegrin and metalloproteinase with thrombospondin motifs) play a significant role. MMPs are a family of zinc-dependent endopeptidases that are capable of degrading the PNN structure. From the over 20 members of MMPs, MMP-1–3, 7–17, 24, and 28 are expressed in the brain. MMP-9 is the most studied endopeptidase in the brain, being expressed by neurons and glial cells [65]. Several studies have demonstrated that MMP-9 expression is upregulated under conditions with higher neural plasticity or activity [66,67,68]. Furthermore, enhanced MMP-9 activity has been observed in several pathological conditions, for example, epilepsy, traumatic brain injury and focal cerebral ischemia [69,70,71,72]. Another group of enzymes involved in PNN remodeling is ADAMTS. ADAMTS forms a family of metalloproteinases that share structural similarities with MMPs [73]. The proteinases ADAMTS-1, 4, 5, 9, and 15 are expressed in the brain. They are capable in degrading CSPGs, mainly aggrecan and brevican [74,75,76,77]. Moreover, alterations in their expression levels have been associated with acute CNS injuries, such as stroke and spinal cord injury [77,78,79]. In addition to changes in the expression levels of MMPs and ADAMTS due to pathologies, their activity can also be internally regulated by molecules known as TIMPs (tissue inhibitors of metalloproteinases). TIMPs were shown to contribute to the restriction of ECM proteolysis by inhibiting the proteolytic activity of MMPs and ADAMTS [80]. Taken together, PNNs are dynamic structures that undergo certain remodeling processes in both physiological and pathological conditions.

## 5. Function of PNNs

Continuous scientific research adds to the growing list of physiological functions assigned to PNNs in the mammalian brain.

### 5.1. Stabilization of Synaptic Sites and Regulation of Synaptic Transmission

**Figure 3:**
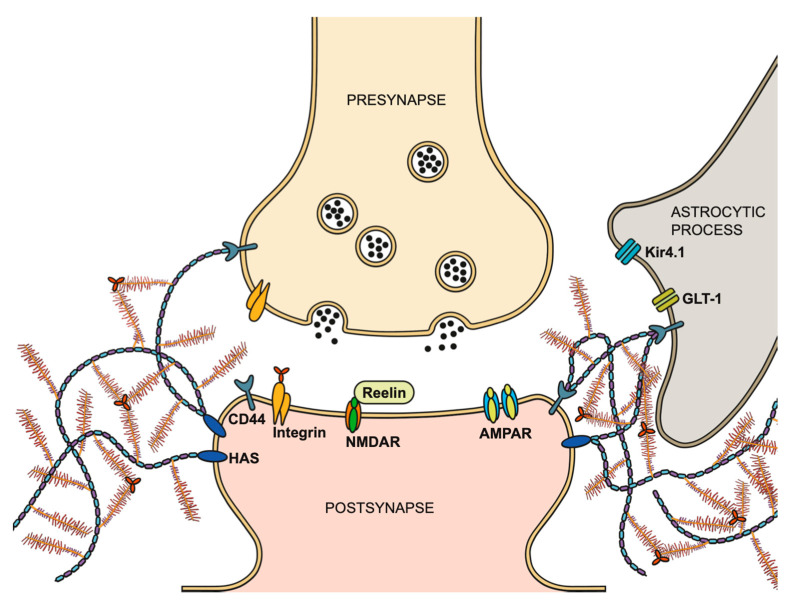
Schematic illustration of the tetrapartite synapse concept. In addition to the presynaptic terminal, postsynaptic terminal, and adjacent astrocytic process, the ECM plays a critical role in modulating synaptic transmission. The presynaptic terminal (yellow) contains CD44 and integrin receptors within its membrane and releases neurotransmitters via synaptic vesicles into the synaptic cleft. At the postsynaptic site (orange), neurotransmitter receptors, such as glutamate receptors NMDAR and AMPAR, are present. The enzyme HAS produces the HA backbone to which lecticans are attached, and these lecticans are linked in triads via TNR. Integrin receptors in the postsynaptic membrane are hypothesized to interact with TNR, while reelin modulates synaptic plasticity through its interaction with NMDAR. The astrocytic process (gray) contains the potassium channel Kir4.1 and the glutamate transporter GLT-1 and features the CD44 receptor, which anchors the HA backbone [81,82,83,84,85]. (Illustration by Jörg Pekarsky, Institute of Functional and Clinical Anatomy, FAU Erlangen-Nürnberg).

Above all, they play a crucial role in the stabilization of synaptic contacts by forming a mesh-like matrix around the cell bodies and proximal dendrites of neurons. The structural framework provided by the PNNs includes “holes” that offer specific sites for synaptic contacts, ensuring the proper functioning of synaptic transmission by restricting the reorganization of the transmission machinery [81]. Recent studies utilizing high-resolution and 3D electron microscopy further elucidated the role of PNNs in synaptic stabilization. The studies have revealed that the “holes” within PNN-surrounded neurons are occupied by synaptic contacts, highlighting the critical role of PNNs in maintaining synaptic contact sites [82]. Beyond the pre- and postsynaptic compartments and the nearby astrocytes, the ECM plays a significant role in physiological synaptic transmission [83]. A study conducted by Blümcke and colleagues in 1995 provided early evidence of the interaction between astrocytic processes and PNNs [84]. Recent advances by Tewari and colleagues (2024) further emphasize this by demonstrating the functional synergy between PNNs and astrocytic processes within the somatosensory cortex of adult mice, underscoring the important role of PNNs in sustaining physiological synaptic function [85]. Collectively, these observations reinforce the concept of the tetrapartite synapse, where the ECM, particularly PNNs, acts as a key participant in structural and functional organization of synaptic networks (Figure 3). The neurotransmitter receptors at the postsynaptic site are highly dynamic in the neuronal plasma membrane [86,87]. The mobility of AMPA-type-glutamate receptors (α-amino-3-hydroxy-5-methyl-4-isoxazolepropionic acid-receptors, AMPAR) represents one example for the importance of lateral diffusion in terms of modulating synaptic transmission. It was shown that PNNs can restrict this lateral diffusion, which demonstrates the control of the availability of synaptic receptors [7]. Moreover, the degradation of hyaluronan results in disruption of the perisynaptic ECM followed by a restoration of the juvenile mobility [7]. This gave rise to the assumption that the ECM plays an important role in modulating synaptic plasticity. In addition to the postsynaptic AMPAR, integrin receptors interacting with ECM components were shown to contribute to long-term potentiation (LTP) by modulating NMDA receptor (N-methyl-D-aspartate receptor, NMDAR) currents [88]. Furthermore, their interaction with the surrounding actin cytoskeleton provides stability [89,90,91,92]. The pre- and postsynaptic hyaluronan receptor CD44 is involved in functional and structural plasticity of dendritic spines, as well as in synaptic stabilization [93]. Another important factor influencing synaptic plasticity is the ECM protein reelin. It was shown that reelin regulates NMDAR and thereby impacts LTP responses [94]. For a more detailed description of the synaptic extracellular matrix, please refer to the review by Dankovich and Rizzoli [81]. Taken together, the ECM has a major impact on the stabilization of synaptic sites and in maintaining proper synaptic transmission.

### 5.2. Regulation of Neural Plasticity

Since PNNs influence the synaptic sites in the CNS, it is reasonable to assume that PNNs contribute to the regulation of neural plasticity. Neural plasticity is a fundamental mechanism driving learning, memory, and cognitive processes, which encompasses the brain’s ability to adapt and reorganize in response to experiences and changes in the environment. These changes rely on the dynamic nature of the PNN structure. The impact of PNNs on neural plasticity becomes particularly prominent during critical periods of various stages of life. These critical periods represent windows of enhanced neural plasticity during which neural circuits exhibit considerable structural and functional changes in response to sensory experiences. Crucially, PNNs exert control over the closure of these critical periods. It was demonstrated that raising animals in complete darkness from birth prevents the formation of PNNs in the adult visual cortex. Moreover, following chondroitin sulfate proteoglycan (CSPG) degradation in adult rats, the ocular dominance plasticity was restored [60]. Additionally, De Vivo et al. showed that enzymatic degradation of CSPG leads to more motile cortical spines with a higher structural and functional plasticity in the adult visual cortex [95]. PNNs were shown to be responsible for the transition from juvenile to adult forms of learning in sensory systems. In adult mice, PNNs provided protection against fear memory extinction. Consequently, removal of PNNs in adults leads to faster erasure of fear memory [56]. Moreover, auditory learning is limited in adult mammals; however, auditory cortex-dependent relearning can restore the agility of the juvenile state following ECM digestion [96]. Collectively, these findings illustrate the impact of PNNs on neural plasticity and thus the role of PNN in orchestrating the closure of critical periods in neural development.

### 5.3. Protection Against Oxidative Stress

Parvalbumin-positive interneurons exhibit fast-spiking properties. To maintain this high neuronal activity, a high metabolic activity is required, which leaves them vulnerable to oxidative damage. PNNs possess highly charged structures with polyanionic characteristics, allowing them to directly influence the local ion homeostasis of the enwrapped neurons and thus providing neuroprotective effects [12]. Suttkus and colleagues demonstrated that their ability to bind redox-active molecules result in a reduction in the local oxidative potential. When analyzing the brains of mice post-injection of FeCl_3_, a significant reduction in neuronal degeneration was observed in neurons surrounded by PNNs compared to KO-mice lacking various PNN components. The findings display that the neuroprotective effects of PNNs are mediated by both the polyanionic charge and the proper interaction of the PNN components [97]. Moreover, the interaction between PNN-ensheathed parvalbumin-positive interneurons and oxidative stress was investigated in mice carrying a genetic redox dysregulation. Oxidative stress profoundly affects parvalbumin-positive cells with immature or impaired PNNs, while parvalbumin-positive cells surrounded by intact PNNs were shown to be protected against excessive reactive oxygen species (ROS) production [11]. Lipofuscin is generated via iron-catalyzed oxidative processes with an accumulation that can be observed in aging and Alzheimer’s disease. PNNs in the human cerebral cortex were shown to provide protective effects against lipofuscin accumulation [12]. Taken together, these finding demonstrate that PNNs exhibit a neuroprotective effect by shielding neurons from oxidative stress and promoting neuronal survival.

### 5.4. Regulation of Neural Circuity Activity

The central nervous system (CNS) contains neurons that exhibit different inhibitory, excitatory, or modulatory characteristics, depending primarily on their respective neurotransmitters. These neurons play a crucial role in regulating neural network activity by maintaining a balanced state of inhibition and excitation, thereby ensuring proper network functioning. In the cerebral cortex, excitation is provided by glutamatergic cells and inhibition is implemented by GABAergic interneurons. Thereby, GABAergic interneurons play a crucial role in regulating the excitatory/inhibitory (E/I) balance and preventing hyperexcitability, which in turn can lead to epilepsy [98]. Due to their positioning around primarily parvalbumin-positive interneurons, PNNs possess the ability to regulate the balance between excitation and inhibition. Moreover, an interneuron dysfunction was shown to be implicated in a variety of psychiatric disorders, including schizophrenia (SZ), autism spectrum disorder (ASD), and bipolar disorder (BPD), and in neurodegenerative disorders, like Alzheimer’s disease (AD), and aging (for a detailed review, see [99]). Parvalbumin-positive interneurons, and thus PNNs, play a critical role in regulating and sustaining gamma oscillations owing to their distinctive inhibitory properties and their involvement in synchronizing neural firing patterns. Gamma oscillations, typically occurring at frequencies between 30 and 90 Hz, are rhythmic patterns of neural activity. These oscillations are integral to cognitive processes, like the formation of memories and the procession of sensory information (reviewed in [100,101]). Consequently, disturbances within gamma oscillations might be linked to psychiatric disorders like schizophrenia [102]. The research on the impact of depletion of PNNs on neural circuit activity mainly involved two approaches: enzymatic degradation using chondroitinase ABC (ChABC) treatment and genetic knockdown of PNN components. The main findings is that the absence of PNNs resulted in a decline in the high firing rate of parvalbumin-positive interneurons (for a detailed overview, refer to [9]).

## 6. Scientific Methods to Study PNNs in the Mammalian Brain

### 6.1. Immunohistochemistry and Imaging Techniques

**Figure 4:**
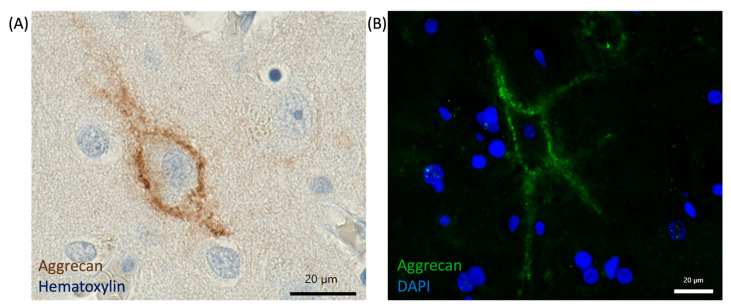
Representative visualization of aggrecan-based PNNs using immunohistochemistry (IHC) and immunofluorescence (IF). (**A**) Immunohistochemical detection of aggrecan-based PNNs in human occipital lobe cortical tissue, stained in brown using rabbit-anti-aggrecan antibody (Proteintech, #13880-1-AP, 1:300), followed by incubation with goat-anti-rabbit-biotinylated secondary antibody (Invitrogen, #31820, 1:200) and DAB substrate reaction. Tissue was counterstained with hematoxylin for visualization of cell nuclei. (**B**) Immunofluorescent staining of aggrecan (green) in human occipital lobe cortical tissue, with DAPI staining (blue) marking cell nuclei, revealing the distribution of PNNs in the cortex. PNNs were detected using rabbit-anti-aggrecan antibody (Proteintech, #13880-1-AP, 1:200), followed by incubation with chicken-anti-rabbit-A488 secondary antibody (Invitrogen, #A21441, 1:1000). Cell nuclei were stained using DAPI (1:1000). Scale bar = 20 µm.

A common technique for visualization of PNNs is the use of the plant-derived lectin *Wisteria floribunda* agglutinin (WFA), which binds to GalNAc present in chondroitin sulfate proteoglycans (CSPGs) [103]. The incubation of tissue sections or cultured cells with WFA conjugated to biotin or a fluorophore allows for a visualization of PNNs. Of note, not all PNNs can be visualized by using lectin detection, due to structural variation in chondroitin sulfate chains, as well as regional variability [21,104]. Instead, antibodies against CSPG components, mainly aggrecan, can be used in immunohistochemical or immunofluorescence approaches [4,24,105] (Figure 4). The analysis with antibodies is much more robust than WFA, supported by the fact that treatment with ChABC cannot abolish the reactivity with an antibody targeted against aggrecan [21,106]. This indicates that there might be a structural heterogeneity of PNNs. Therefore, it is recommended that various markers should be used, to explore this heterogeneity when studying PNNs. Fluorescence microscopy allows for visualizing PNNs labeled with either lectins or antibodies conjugated to a fluorophore. This technique was used in various studies and offers high-resolution imaging of the PNN distribution and structure within tissue sections (Figure 4). By using double or triple staining, researchers can assess the colocalization of different molecular components and investigate the cellular subtypes enwrapped by PNNs [107,108,109,110,111]. Transmission electron microscopy (TEM) can be used to gain an even deeper insight into PNNs. TEM provides ultrastructural details of PNNs in the nanometer range [82,112,113]. This enables visualization of the fine structure of PNNs, as well as their interaction with surrounding neuronal elements and other extracellular matrix components. In combination with immunogold labeling or DAB labeling, TEM permits selective localization and analysis of various PNN components. These immunohistochemistry and imaging techniques offer valuable tools in the research on PNNs and provide insight into their distribution, structure, and role in neural development, plasticity, and disease.

### 6.2. Knocking out Different PNN Components in Rodents

Various studies in rodents using knock-out techniques have been performed to specifically target and understand the function of each PNN component. In 2010, Giamanco and others studied the role of aggrecan in PNN formation by using aggrecan-deficient *cmd* mice (cartilage matrix deficiency in mice). The evaluation of neuronal cultures as well as organotypic slice cultures in immunohistochemistry (IHC) or immunocytochemistry (ICC) approaches showed that staining with the lectin *Wisteria floribunda* agglutinin (WFA) was absent in mice lacking aggrecan. On the contrary, the other components hyaluronan and proteoglycan link protein 1 (HAPLN1), brevican, hyaluronan (HA), and tenascin R (TNR) remained unaffected. Additionally, the binding of HAPLN1 to the cell surface was still intact after treatment with the enzyme chondroitinase ABC, indicating hyaluronan-dependent binding. In contrast, brevican, TNR, and HA were shown to be bound to the cellular surface in an hyaluronan- and CS-dependent manner [114]. In a recent study by Rowlands and colleagues, the aggrecan gene *Acan* was genetically deleted in an established *Acan*-IoxP mouse model. This resulted in a reactivation of the critical-period plasticity in the visual cortices of adult mice, suggesting a major role of aggrecan in PNN formation, plasticity, and memory function [115]. The PNN component brevican was also studied using knocking-out techniques. Brevican KO mice showed a normal induction of long-term potentiation (LTP) but had deficits in the maintenance of hippocampal LTP [116]. Favuzzi and coworkers demonstrated that brevican is an essential regulator of interneuron plasticity. This was substantiated by the ability of brevican to control the localization of potassium channels and AMPA-type receptors [117]. The role of neurocan in PNN development has been studied by using neurocan-deficient mice [118]. The medial nucleus of the trapezoid body (MNTB) in the auditory brainstem was analyzed via immunohistochemical approaches. In conclusion, the study provided evidence that neurocan controls the regulation of PNN development by influencing the mRNA and protein quantity of various PNN components in the MNTB [118]. In addition to the study of CSPGs, the role of TNR was also analyzed in knock-out studies. Mice deficient in TNR showed an attenuated PNN structure, implying the crucial role of TNR in both formation and stabilization of PNNs by clustering with aggrecan [119]. To understand the basis of the neuroprotective effect of PNNs, four different mice strains lacking PNN components were treated with FeCl_3_ to induce neurodegeneration. Aggrecan, link protein, and TNR were identified as essential in maintaining the neuroprotective effect of PNNs, whereas the contribution of brevican was insignificant [97]. Knocking out the link protein CRTL1 resulted in a weakened PNN structure and a retention of the critical period visual plasticity. The expression of other PNN components was not altered [61]. The link protein BRAL2 was shown to be indispensable for proper brevican localization, indicating a pivotal role in the organization of PNNs [120]. In a recent study, the interaction of TNR and RPTPζ/phosphacan was studied by comparing the PNN phenotype of *Tnr* −/− and *Ptprz1* −/− mice. Both displayed a disrupted PNN structure indicating a cooperation of TNR and RPTPζ/phosphacan in the proper assembly of PNNs [121]. This interaction was also proven by a study of Eill and others, analyzing the role of the protein tyrosin phosphatase RPTPζ/phosphacan in *Ptprz1* −/− mice [35]. In studies using quadruple KO mice lacking TNC, TNR, brevican, and neurocan, PNNs were analyzed. The results of the studies displayed a reduction in PNN-sheathed cells and a disrupted PNN structure. Moreover, the ratio of inhibitory and excitatory synapses was imbalanced, with a reduction in inhibitory synaptic elements within PNNs. In addition, the number of parvalbumin-positive cells was reduced in the KO mice. Finally, the transcription factor OTX2 (orthodenticle homeobox 2) was shown to be decreased. Overall, this confirms that TNR, TNC, brevican, and neurocan play a significant role in modulating the dynamic interaction among PNNs, synaptic integrity, inhibitory neurons, and the transcription factor OTX2 [122,123].

### 6.3. Enzymatic Degradation of PNN Components (ChABC, Hyaluronidase)

In addition to the genetic ablation of PNN components, enzymatic degradation of PNNs offers a further, more locally applicable option for investigating the influence of PNN changes. The enzymatic disruption of PNNs involves the application of enzymes to degrade molecular components of PNNs. This provides a valuable tool for studying the functional consequences on neural function or plasticity following PNN alterations. The enzyme chondroitinase ABC (ChABC) is commonly used for this purpose. ChABC induces the ablation of PNNs by enzymatic degrading of sugar chains (glycosaminoglycans or GAGs) of CSPGs. The efficacy of this ability can be validated through the elimination of WFA staining, which is a commonly used marker for PNNs [124]. The enzymatic manipulation of PNNs was used in various studies (reviewed in [125]). In a recent study, the importance of PNNs in the hippocampus and cortex in terms of contextual fear memories was shown [126]. Hence, the elimination of PNNs through ChABC administration led to impaired memory consolidation. In recent work by Willis and coworkers, the disruption of PNNs via ChABC administration facilitated juvenile-like plasticity by enhancing structural flexibility [127]. Further, treating parvalbumin-positive interneurons in the primary visual cortex with ChABC resulted in changes in neuronal excitability and synaptic transmission [128]. Within the hippocampal CA1 region, removal of PNNs via ChABC injection led to a reduced firing rate of parvalbumin-positive neurons and decreased synaptic transmission. Moreover, ChABC treatment interfered with the maintenance of long-term contextual fear memory [129]. In mice lacking CRTL1, administration of ChABC made fear memories more susceptible to erasure, suggesting a role of PNNs in regulating the boundaries of the critical period for fear extinction [130]. Another opportunity to disrupt the structure of PNNs enzymatically arises with the application of the enzyme hyaluronidase. Hyaluronidase cleaves the HA backbone of the PNNs. In one study, it was shown that the disruption of PNNs via HA treatment had an impact on the neurophysiology of hippocampal cells [131]. However, the enzymatic degradation of PNNs using either ChABC or hyaluronidase displays several limitations; for example, the digestion cannot be specifically targeted to PNN-ECM. Here, the recently developed Cre-LoxP system by Carstens and coworkers offers an alternative tool. It allows for cell-type-specific targeting and for longer-lasting ChABC degradation [132]. This system can be used to further study the effect of the durable loss of PNNs and to identify strategies to potentially treat neurological disorders.

### 6.4. Proteomic Studies

A novel approach for studying the composition of the brain’s ECM is proteomic analysis. In this way, the use of mass spectrometry allows for the identification and quantification of the proteins present in the tissue and provides valuable insights into the role of ECM alterations in neurological diseases. Recent studies have employed this approach to uncover differences in the proteome in epilepsy. For instance, proteomic differences could be identified in the hippocampus and cortex of epilepsy brain tissue, displaying significant differences in the expression of 777 proteins in the hippocampus and 296 proteins in the cortex, indicating that proteins associated with protein synthesis, mitochondrial function, G-protein signaling, and synaptic plasticity are altered in epilepsy [133]. However, this study did not specifically target ECM-associated proteins. In studies focusing on the ECM, proteomic analyses in mice and human revealed a link between ECM alterations and cerebrovascular diseases [134]. In addition, the identification of the proteomic profile of ECM proteins in aging and stroke could reveal an increased activation of genes encoding proteins related to ECM remodeling. Moreover, CSPGs, syndecans, and link proteins were upregulated in aged murine models [135]. In a recent study by Leitner and colleagues, the proteomic signatures of Alzheimer’s disease (AD) and epilepsy were compared, demonstrating significant overlap in the differentially expressed proteins involved in synaptic and mitochondrial dysfunction [136]. Finally, a study by Srivastava and colleagues analyzed the molecular basis for mesial temporal lobe epilepsy (MTLE). By employing proteomics and protein co-expression network analysis, the study analyzed brain tissue from patients with MTLE and revealed altered protein networks and pathways. These disruptions were linked to pathways such as synaptic vesicle neurotransmitter release, synaptic plasticity, metabolic and mitochondrial dysfunction, as well as extracellular matrix organization and cell signaling. This study provided insights into the pathogenesis of MTLE. The advances in proteomics in epilepsy alongside discussions of mass spectrometry methods for analyzing ECM components in neurological disorders have been reviewed in various papers [137,138]. While many recent studies have not prioritized ECM-specific proteins, ECM-targeted proteomic approaches offer valuable tools for investigating alterations in the ECM composition and provide insights into the role of ECM remodeling under pathological conditions.

## 7. PNNs and Their Implications for Neurological Disorders

### 7.1. Schizophrenia and Bipolar Disorder

Schizophrenia (SZ) represents a multifaceted psychiatric disorder, marked by a combination of positive symptoms, like hallucinations or delusions, negative symptoms, such as deficits in the expression of emotions, as well as cognitive impairments. Recent research suggests a potential link between PNNs and the pathophysiology of schizophrenia. Post-mortem studies have revealed abnormalities in the ECM in brain regions like the amygdala and entorhinal cortex. Specifically, individuals with schizophrenia exhibited an increase in CSPG-positive glial cells and a reduction in PNN density in the lateral nucleus of the amygdala and layer II of the entorhinal cortex. These findings underscore the significance of the interaction of the ECM and glial cells in the development of schizophrenia [139]. Moreover, post-mortem studies of the olfactory epithelium revealed differences in the expression of CSPGs [140]. Analysis of post-mortem human brains with schizophrenia showed a reduction in PNN density in layers III and V of the prefrontal cortex [141]. However, PNN density remain unchanged in the primary visual cortex. These findings suggest that PNNs might contribute to the dysfunction of the prefrontal cortex seen in schizophrenia. Additionally, the timing of PNN development coincides with the emergence of schizophrenia symptoms, implying a potential role for PNN formation in the onset of the illness [141]. Abnormalities of aggrecan and chondroitin-6-sulfate were examined in a study by the research group around Pantazopoulos. The use of post-mortem tissue from the amygdala of schizophrenia patients showed that the number of aggrecan PNNs was decreased, and CSPG abnormalities could be observed [105]. In the dorsolateral prefrontal cortex, the density of parvalbumin-positive cells remained unchanged in individuals with schizophrenia. However, the fluorescence intensity of parvalbumin labeling was notably lower in comparison to controls. In addition, there was a decrease in the immunoreactivity of PNNs positive for WFA and aggrecan in the schizophrenia group. These observations suggest distinct differences in the levels of parvalbumin protein and PNN components among individuals with schizophrenia [111]. In an experimental model of schizophrenia using ketamine-treated rats, researchers investigated the ECM expression. A reduced number of WFA-labeled PNNs and a decrease in PV fluorescence intensity were observed in interneurons within the prefrontal cortex [142]. In the thalamic reticular nucleus of transgenic mice with a redox dysregulation, the numbers of parvalbumin-positive neurons and PNNs were decreased in the rodent model for schizophrenia and bipolar disorders compared to wild-type (WT) mice [143]. Another mouse model used for studying schizophrenia is *Gclm* KO mice with a genetically compromised glutathione system. Here, a deficit in parvalbumin neurons was found due to their high susceptibility to oxidative stress [144]. By using RNA sequencing approaches, the transcriptome revealed differences of more than 25% in differential splicing or expression levels in subjects with autism spectrum disorder (ASD), schizophrenia, and bipolar disorders (BPDs) [145]. Post-mortem sections of BPD patients revealed a significantly lower density of PNNs in the dorsolateral prefrontal cortex, whereas schizophrenia patients showed only a slight reduction in PNN density in this brain region [146]. PNN remodeling by endopeptidases was also shown to be affected in schizophrenia, as indicated by a dysregulation of MMP-9 expression [147]. In more recent studies using the ketamine mouse model of schizophrenia, treated mice exhibited fine structure abnormalities and a quantitative reduction in PNNs [148,149]. Analyzing the molecular signature with microarray analysis, as was examined in a study by Pantazopoulos and colleagues, revealed a differential expression of several ECM components, e.g., brevican, neurocan, and a disintegrin and metalloprotease with thrombospondin motifs 1 (ADAMTS1), in schizophrenia [150]. In the prefrontal cortex of a schizophrenia mouse model (disruption in schizophrenia, DISC1), both PNNs and parvalbumin-positive neurons were decreased. Following digestion with ChABC, the behavior of the control mice matched that of the DISC1 mice. Moreover, the number of high-frequency firing neurons was reduced, whereas the ratio of irregularly firing neurons was increased [151]. In a recent study by Liang and Zhang, the effects of an inhibition of NMDAR in young-aged mice, combined with social stress in adulthood, were assessed. The number of PV+ cells enwrapped by PNNs was increased, and the activity of PV+ neurons was decreased in the prefrontal cortex in mice with an inhibition of NMDAR [152]. Overall, substantial evidence indicates the crucial role of PNNs in the pathophysiology of schizophrenia and bipolar disorders (for a review, see [153,154,155,156,157]).

### 7.2. Alzheimer’s Disease (AD)

Alzheimer’s disease is a progressive, neurodegenerative disease and represents the most common underlying cause of dementia. The symptoms include successive memory impairment, changes in behavior and personality, and difficulties in reasoning, language, and handling of complex tasks. The underlying reason for AD is not yet fully understood. However, the abnormal accumulation of proteins such as amyloid plaques and Tau protein tangles in the brain can lead to the death of brain cells. To date, there is no curative treatment option for AD available. Yet, some medications can slow the progression of dementia symptoms. Immense efforts are being made to research the causes of AD and to find novel treatment options. Several studies have investigated the involvement of PNNs in the pathophysiology of AD. In a study using samples of AD patients, it was shown that neurons, which are enwrapped by aggrecan-based PNNs, displayed protection against the accumulation of Tau fibers. In regions that were heavily affected by Tau pathology, aggrecan-based ECM was absent. This suggests that the aggrecan ECM may contribute to the protection of neurons from Tau protein accumulation in AD [158]. On the contrary, in an Alzheimer model using transgenic *Tg2576* mice, PNNs were largely unaffected when investigating aggrecan-based PNNs. However, with advanced progression of the disease, PNNs were removed after cell death [159]. By detecting the main components of PNNs in temporal and occipital lobes of AD patient tissue, a study by Morawski and colleagues confirmed that aggrecan-based PNNs provide protection against Tau pathology. Conversely, the PNN structure and distribution remained unchanged within the amyloid pathology in AD tissue [160]. In the hippocampi of AD patients, loss of PNNs was observed. Additionally, the formation of axonal coats was shown to contribute to the preservation of synaptic integrity within AD-related change [109]. Several studies conducted proteome analysis to investigate protein alterations in AD. Various ECM proteins were shown to be dysregulated within AD conditions [157]. In a novel mouse model *TauP301L*, which lacks aggrecan, high levels of Tau protein were observed [161]. Using mass spectrometry analysis, a change in the sulfation patterns of the PNN-associated CS-GAGs was observed in patients with AD. These findings correlate with AD progression and with Tau accumulation, as well as with the impairment of cognition [162]. A possible neuroprotective function of aggrecan and other PNN components was demonstrated in a study using a Tau/aggrecan double transgenic model [163]. Studying the proteomic signature of AD and epilepsy tissue revealed overlapping changes in the protein composition, with 89% of the proteins altered in the epileptic hippocampus also shown to undergo changes in AD patients [136]. In summary, PNNs may have a substantial role in the development and progression of AD and potentially offer protective effects in relation to the disease’s pathology. This suggests a potential of compounds targeting PNNs as a promising therapeutic strategy for treating AD (for a review, see [15]).

### 7.3. Epilepsy

Epilepsy is a chronic neurological disorder marked by recurrent, spontaneous seizures, affecting approximately 50 million people worldwide [164]. The main cause is an imbalance in the electrical activity of the brain. Given that PNNs are involved in regulating and maintaining the balance between excitation and inhibition, as well as in regulating the excitability of PV+ neurons, it is plausible to assume that PNNs are involved in the pathogenesis of epilepsy. Moreover, by modulating synaptic plasticity, PNNs are implicated in the pathophysiology of epilepsy. Numerous studies investigated the ongoing remodeling of the ECM in epileptic brain tissue. In a rodent model of temporal lobe epilepsy (TLE), PNN components were analyzed following status epilepticus (SE). A persistent decrease in PNN components including aggrecan, hyaluronan and proteoglycan link protein 1 (HAPLN1), and hyaluronan synthetase 3 (HAS3) was detected in the epileptic hippocampus [165]. The degradation of PNNs was analyzed in an epilepsy rodent model in a study by Rankin-Gee and colleagues [70]. By inducing SE, the MMP proteolysis of aggrecan was increased. Moreover, a loss of PNNs was associated with a higher number of seizures [70]. Further studies showed that MMP activity, in particular, MMP-3 and MMP-13, was increased in the hippocampi of rats after the induction of SE. In contrast, the activity of ADAMTS was not significantly increased after status epilepticus [166]. Thus, these studies provide evidence that the PNN remodeling in epilepsy is primarily driven by MMP activity. In epilepsy associated with brain tumors, the ability of PNNs to modulate the firing frequency of cells has been demonstrated using rodent models. PNNs were shown to be able to reduce the membrane capacity of fast-spiking interneurons, letting them fire action potentials in a more than physiological manner. Therefore, the loss of PNNs led to a reduced firing rate of fast-spiking interneurons, which represents the underlying cause of tumor-associated seizures [167]. A study comparing the developmental timeline of PNNs during aging suggested no differences between controls and epilepsy patients. However, this conclusion is limited due to the small sample number of epileptic surgical specimens examined and the inclusion of different epilepsy types, leaving the findings inconclusive [57]. In contrast, a more recent study observed an age-dependent increase in PNNs in the human hippocampus [24]. Additionally, precocious aging effects could be determined in TLE patients with hippocampus sclerosis, characterized by a higher PNN density in immunohistochemical analyses of surgical specimens. This increase was not accompanied by an increase in the PV+ neuronal cell density, raising questions about the source of PNN material and their target cell population. These observations corroborated, however, that PNNs are involved in the formation of memory engrams in the human hippocampus [24]. In summary, these studies underscore the role of PNNs in the pathophysiology of epilepsy by modulating synaptic plasticity and neuronal excitability. The ongoing PNN degradation is predominantly driven by increased MMP activity and a higher seizure frequency. Moreover, age-dependent changes in PNN density could be found in the epileptic hippocampus. Consequently, targeting PNNs with drugs may hold potential as a therapeutic intervention in epilepsy (for a detailed review, see [14]).

## 8. Therapeutic Potential and Future Directions

The integrity of the ECM, particularly PNNs, plays an important role in regulating synaptic plasticity and maintaining neuronal excitability. Recent studies have increasingly highlighted the role of the ECM in the pathogenesis of various diseases of the CNS. Consequently, targeting ECM components could represent a novel treatment option for neurological disorders.

In epilepsy, the ongoing ECM remodeling is mainly driven by MMP activity. Hence, inhibiting MMP enzymatic activity might offer a promising option for treating epilepsy. By employing doxycycline as an MMP inhibitor, PNN degradation could be prevented, and seizure occurrence could be reduced in an epilepsy rodent model [168]. Similarly, the MMP inhibitor IPR-179 demonstrated antiseizure effects in a rat temporal lobe epilepsy model. The seizure intensity could be reduced by IPR-179 treatment without severe side effects. These findings suggest the potential of MMP inhibition as a promising therapeutic approach against epilepsy, though further investigation is needed [169].

In the context of AD, PNNs may represent a promising target, as they are involved in memory and cognition. Manipulating the PNNs could reactivate plasticity and restore memory functions impaired in AD pathogenesis. Various strategies targeting PNNs have been proposed, including enzymatic degradation, genetic therapy to inhibit PNN formation, and the use of compounds or molecules to block PNNs [170]. However, it is also important to consider the protective effects of PNNs in preventing the accumulation of Tau or amyloid-beta proteins in AD pathology (see Section 7.2). This protective role could have a beneficial effect in slowing the progression of the disease.

While therapeutic approaches targeting PNNs in neurological disorders like epilepsy and AD appear promising, the precise role of PNNs in the pathophysiology of these and other neurological diseases is not yet fully understood. Further research is, therefore, needed to elucidate the molecular mechanisms by which PNNs contribute to the pathogenesis. In addition, further studies should focus on improving the methods used to study PNNs in their physiological state, which is critical for gaining a deeper understanding of PNN dynamics and functions. Understanding PNNs under normal conditions is essential to better understand how alterations might contribute to neurological disorders, and thus, to explore potential therapeutic interventions.

## Data Availability

No new data were created or analyzed in this study. Data sharing is not applicable to this article.

## Abbreviations

The following abbreviations are used in this manuscript:

PNN	Perineuronal net
CNS	Central nervous system
ECM	Extracellular matrix
PV+	Parvalbumin expressing
GABA	Gamma-aminobutyric acid
HA	Hyaluronic acid
CSPG	Chondroitin sulfate proteoglycan
CA	Cornu ammonis
BA	Brodmann area
TNR	Tenascin R
CRTL1	Cartilage link protein 1
HAPLN	Hyaluronan and proteoglycan link protein
BRAL2	Brain link protein 2
CD	Cluster of differentiation
HAS	Hyaluronan synthase
GAG	Glycosaminoglycan
GlcA	Glucoronic acid
GalNAc	N-acetlyl-galactosamine
RPTP	Receptor protein tyrosine phosphatase
SEMA3A	Semaphorin 3A
OTX2	Orthodenticle homeobox 2
MMP	Matrix metalloproteinase
ADAMTS	A disintegrin and metalloproteinase with thrombospondin motifs
TNC	Tenascin C
TIMP	Tissue inhibitor of metalloproteinases
AMPAR	α-amino-3-hydroxy-5-methyl-4-isoxazolepropionic acid receptor
NMDAR	N-methyl-D-aspartate-receptor
LTP	Long-term potentiation
ROS	Reactive oxygen species
E/I	Excitatory/inhibitory
SZ	Schizophrenia
ASD	Autism spectrum disorder
BPD	Bipolar disorder
AD	Alzheimer’s disease
ChABC	Chondroitinase ABC
WFA	*Wisteria floribunda* agglutinin
TEM	Transmission electron microscopy
DAB	3,3′-diaminobenzidine
IHC	Immunohistochemistry
IF	Immunofluorescence
DAPI	4′,6-diamidin-2-phenylindol
Cmd	Cartilage matrix deficiency
ICC	Immunocytochemistry
KO	Knock-out
MNTB	Medial nucleus of the trapezoid body
MTLE	Mesial temporal lobe epilepsy
SE	Status epilepticus
TLE	Temporal lobe epilepsy
